# Association between urinary nickel with obesity status in adults: A cross-sectional study

**DOI:** 10.3389/fpubh.2023.1094062

**Published:** 2023-02-17

**Authors:** Gao-Xiang Wang, Bao-Li Huang, Jun-Tong Li, Ze-Bin Fang, Le-Yi Feng, Heng-Xia Zhao, Shu-Fang Chu, De-Liang Liu, Hui-Lin Li

**Affiliations:** ^1^Shenzhen Traditional Chinese Medicine Hospital Affiliated to Nanjing University of Chinese Medicine, Shenzhen, Guangdong, China; ^2^Department of Endocrinology, Shenzhen Traditional Chinese Medicine Hospital, Shenzhen, Guangdong, China; ^3^The Fourth Clinical Medical College of Guangzhou University of Chinese Medicine, Shenzhen, Guangdong, China

**Keywords:** urinary nickel, obesity, body mass index, waist circumference, NHANES

## Abstract

**Objectives:**

The prevalence of obesity is on the rise and is connected to numerous factors. However, the relationship between obesity and nickel has never been investigated. Our study aimed to explore the association between urinary nickel and obesity Status in adults.

**Methods:**

From the 2017–2018 National Health and Nutrition Examination Surveys (NHANES), 1,705 participants ≥18 years of age were enrolled. To explore further the relationship among urinary nickel, body mass index (BMI), and waist circumference(WC), Weighted multivariate linear regression analyses and further subgroup analyzes were conducted.

**Results:**

Urinary nickel does not correlate with BMI level but positively correlates with WC. In the subgroup analyzed according to sex, Urinary nickel has a positive correlation with BMI and WC in males but has a negative correlation in females. Secondary stratification analysis according to sex and race, Urinary nickel positively correlates with BMI in White males. It also positively correlates with WC in both White and Black males.

**Conclusions:**

A correlation was found between urinary nickel levels and BMI and WC in adult males. Adult men, especially those already obese, may need to reduce nickel exposure.

## Introduction

Nickel occupies the 28th spot in the periodic table. It is a brutal metal found naturally in air, water, and soil (1). Nickel is vital for microorganisms, plants, animals, and humans (2). Nickel deficiency can cause growth retardation and fecundity decline, impairment of specific senses, reduced iron absorption, and alteration of essential enzymes in animal tissues and organs, leading to various clinical changes (3–5). However, specific toxicity and carcinogenic properties are connected with excessive nickel. In humans, numerous health issues, including contact dermatitis, cardiovascular conditions, and lung and nasal cancer, can result from prolonged exposure to nickel (6). Nickel exposure most commonly occurs through respiratory inhalation (7), food and water intake (8), and skin absorption (9). With the extensive use of nickel-containing products in daily life (10), especially in medical devices (11), great attention is paid to nickel-related health issues.

Globally, obesity has become a severe public health issue (12). Research shows that 70% of American and 50% of Chinese adults are overweight or obese (13, 14). There are several diseases associated with obesity, such as hypertension (15), malignant tumors (16), and diabetes (17). Several investigations conducted during the COVID-19 pandemic also revealed that obese people with COVID-19 infection had much greater rates of severe illness and fatality than normal persons (18, 19). Since obesity constitutes a significant threat to health, a more profound knowledge of relevant factors of obesity is necessary. Recent studies have confirmed a correlation between urinary nickel and the prevalence of diabetes and high blood pressure (20, 21). As everyone knows, diabetes and high blood pressure are closely related to obesity, but the relationship between urinary nickel and obesity status is unclear (22).

Body mass index (BMI) can effectively assess the state of health, has the characteristics of simple, feasible, and non-invasive (23, 24), and is an essential indicator for the diagnosis of obesity. Waist circumference (WC) has become increasingly crucial in predicting death and morbidity in recent years, and the combination of WC and BMI has been emphasized in diagnosing obesity (25, 26). In this study, we conducted a cross-sectional study to explore the relationship between urinary nickel and obesity index (BMI and WC). To our knowledge, this is the first study to examine the relationship between obesity status and nickel exposure, which is of great significance to this field.

## Materials and methods

### Study population

**Figure 1:**
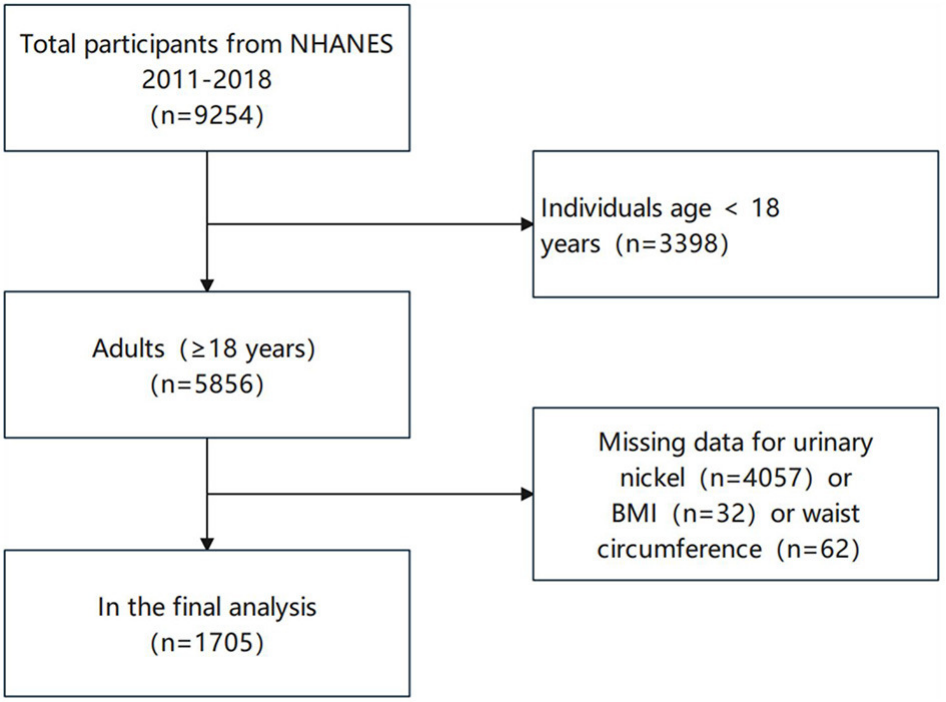
Flow chart for selecting participants.

In this study, our subjects included adults (≥18 years) from NHANES during 2017–2018. Study participants had 1,705 people after eliminating those with missing data regarding urinary nickel, BMI, or WC. The selection process is depicted in Figure 1.

### Ethics statement

Participation in the study was voluntary, and the National Center for Health Statistics Research Ethics Review Board approved this study's conduct. To protect everyone's privacy, NHANES will anonymize collected data before making it public as public data. We agree to follow all guidelines for using NHANES data for research purposes and comply with all applicable standards and laws.

### Urinary nickel, BMI, and WC

It was decided to collect a single spot urine sample and store it at ≤-20°C for long-term or short-term analysis stored at 2–8°C before analysis. Inductively coupled plasma mass spectrometry (ICP-MS) was employed to determine nickel levels in urine, which is a susceptible technique that can measure multiple elements at low concentrations. To be brief, ICPs operate with argon flows passing through an atomizer and spray chamber to process urine samples. The sample vaporizes at a high temperature, dissociates the ionized gas, and then the ions reach the ion detector. Finally, the isotope ratio of the elements is measured. A urinary nickel concentration of 0.31 mg/L is considered a detection limit of detection. More detailed laboratory procedure manuals are shown on the NHANES's website (27).

This study measured the weight, height, and WC of adults 18 and older using standardized methods. Physical examination measured BMI and WC. BMI is calculated by dividing the square of a person's weight (in kilograms) by their height (in meters), and precise measurements could well be acquired using standard digital scales and rulers. Medical professionals measured the subjects' WC with a flexible ruler. According to the WHO-recommended measurement method, the subject's feet were separated by 25–30 cm. The measurer placed the measuring tape around the abdomen in a circle at the midpoint of the line connecting the anterior superior iliac crest and the lower border of the 12th rib, close to the soft tissue, but without compression, and measured at the end of exhalation and before inspiration. When the WC and BMI are both normal, it is not obesity; when the BMI is normal, but the WC of men is ≥94 cm, and that of women is ≥80 cm, it is central obesity; when the WC is standard, but the BMI is ≥30 kg/m^2^, it is defined as peripheral obesity; when the BMI and WC are both above normal, it is defined as mixed obesity (28, 29).

### Covariates

The information on age, race, the ratio of family income to poverty, and the educational level of the participants was obtained through the questionnaire. To ensure the quality of the questionnaire and data collection, questionnaires are developed in advance by professional surveyors and released. Data is received by trained medical staff at a mobile medical examination center. Qualified laboratory specialists collected and processed blood samples at the mobile medical examination center. The following parameters will be evaluated: total cholesterol, triglycerides, glycohemoglobin, blood urea nitrogen, serum creatinine, serum uric acid, and total protein. Hispanic, Mexican American, Non-Hispanic White, Non-Hispanic Black, and Other Race were classified. Education levels below high school, high school, and higher education were ranked based on their education level. The NHANES website (www.cdc.gov/nchs/nhanes/) provides public access to the data from this survey.

### Statistical analysis

This study used statistical software R (version 3.4.4) to conduct all statistical analyzes. Per National Center for Health Statistic (NCHS) recommendations, samples were weighted according to NHANES (30). An analysis of the associations between urinary nickel, BMI, and WC was conducted using weighted linear regression. In this study, we built three regression models. No adjustments had been made to Model 1: race, gender, and age adjustment were made in Model 2. A complete adjustment was made to Model 3 for all Covariates. To further explore the relationship between urinary nickel, BMI, and WC, Weighted multivariate linear regression analyses and subgroup analyzes were conducted. A *p*-value of <0.05 determined statistical significance.

## Results

### Description of participant characteristics

The statistical characteristics of the study population are displayed in Table 1. A total of 1,705 adults participated in this study. In different groups of urinary nickel (quartiles, Q1-Q4), gender, age, triglycerides, serum creatinine, and BMI were not statistically significant. In contrast, race/ethnicity, education level, the ratio of family income to poverty, total cholesterol, glycohemoglobin, blood urea nitrogen, serum uric acid, total protein, WC, and obesity status were statistically significant. The main types of obese people are mixed and central obesity.

**Table 1:** Weighted characteristics of the study sample.

**Urinary nickel**	**Total**	**Q1**	**Q2**	**Q3**	**Q4**	***P*-value**
**Gender (%)**						0.217
Male	48.61	46.87	49.79	52.12	45.36	
Female	51.39	53.13	50.21	47.88	54.64	
Age (years)	47.04 ± 17.54	46.83 ± 15.62	46.92 ± 17.75	46.43 ± 18.07	48.16 ± 18.86	0.554
**Race/ethnicity (%)**						0.010
Mexican American	9.16	8.96	9.68	7.93	10.19	
Other Hispanic	6.37	7.34	5.34	6.57	6.14	
Non-Hispanic White	62.64	63.46	66.64	64.25	54.84	
Non-Hispanic Black	11.32	8.6	10.18	13.12	14.11	
Other race	10.52	11.63	8.17	8.13	14.71	
**Education level (%)**						0.007
Less than high school	11.21	9.73	9.05	12.82	13.87	
High school	27.79	23.85	27.97	32.46	27.21	
More than high school	61.01	66.43	62.97	54.73	58.92	
Ratio of family income to poverty (%)	2.97 ± 1.58	3.29 ± 1.56	2.99 ± 1.59	2.86 ± 1.59	2.67 ± 1.50	<0.001
Total cholesterol (mmol/L)	4.83 ± 1.00	4.94 ± 1.03	4.93 ± 1.00	4.74 ± 1.00	4.68 ± 0.93	<0.001
Triglyceride (mmol/L)	1.59 ± 1.32	1.51 ± 0.94	1.62 ± 1.37	1.59 ± 1.00	1.63 ± 1.88	0.476
Glycohemoglobin (%)	5.67 ± 0.90	5.57 ± 0.68	5.68 ± 1.00	5.72 ± 0.97	5.71 ± 0.93	0.046
Blood urea nitrogen (mmol/L)	5.32 ± 1.84	5.03 ± 1.64	5.22 ± 1.82	5.51 ± 1.76	5.59 ± 2.12	<0.001
Serum creatinine (umol/L)	77.29 ± 23.18	76.29 ± 18.12	76.33 ± 25.40	78.60 ± 18.98	78.21 ± 29.52	0.313
Serum uric acid (umol/L)	319.33 ± 80.89	311.22 ± 79.13	316.48 ± 80.56	326.22 ± 83.56	325.23 ± 79.22	0.016
Total protein (g/L)	71.09 ± 4.21	71.28 ± 3.86	71.53 ± 4.08	70.66 ± 4.42	70.81 ± 4.47	0.008
Body mass index (kg/m^2^)		29.14 ± 6.67	29.73 ± 6.70	30.24 ± 7.26	30.28 ± 7.16	0.053
Waist circumference (cm)		99.09 ± 16.03	100.60 ± 16.44	102.24 ± 18.33	102.51 ± 18.38	0.012
**Obesity status**						0.026
No obesity	23.78	24.50	26.59	20.34	23.41	
Central obesity	32.08	36.89	29.28	33.79	27.39	
Peripheral obesity	0.04	0	0	0.17	0	
Mixed obesity	44.09	38.61	44.14	45.70	49.19	

Continuous variables are presented as Mean ± SD. A weighted linear regression model was used to calculate the *P*-value. A categorical variable is shown as a percentage, and Chi-square test was used to calculate the *P*-value.

### Covariable selection

As shown in Table 2, we select covariates by univariate analysis. When the outcome index is BMI, the age, race/ethnicity, education level, ratio of family income to poverty, total cholesterol, triglyceride, glycohemoglobin, and serum uric acid were select as covariable. When the outcome index is WC, the age, gender, race/ethnicity, education level, blood urea nitrogen, serum creatinine, total cholesterol, triglyceride, glycohemoglobin, serum uric acid, and total protein were select as covariable.

**Table 2:** Univariate analysis to select covariates.

**Urinary nickel**	**Body mass index (kg/m^2^) β (95% CI), *P***	**Waist circumference (cm) β (95% CI), *P***
**Gender**		
Male	Reference	Reference
Female	0.43 (−0.23, 1.09)	−4.13 (−5.76, −2.50)^***^
Age	0.03 (0.01, 0.05)^**^	0.21 (0.16, 0.25)^***^
**Race/ethnicity**		
Mexican American	Reference	Reference
Other Hispanic	−1.39 (−3.08, 0.31)	−3.37 (-7.58, 0.83)
Non-Hispanic White	−0.95 (−2.11, 0.21)	0.57 (−2.31, 3.46)
Non-Hispanic Black	0.10 (−1.36, 1.56)	−0.90 (−4.52, 2.73)
Other race	−3.08 (−4.56, −1.60)^***^	−6.71 (-10.39, −3.03)^***^
**Education leve**		
Less than high school	Reference	Reference
High school	1.94 (0.77, 3.10)^**^	4.20 (1.30, 7.10)^**^
More than high school	1.09 (0.02, 2.16)^*^	1.55 (−1.11, 4.22)
Ratio of family income to poverty	−0.21 (−0.42, −0.00)^*^	−0.08 (−0.60, 0.44)
Total cholesterol	0.45 (0.12, 0.78)^**^	1.48 (0.66, 2.30)^***^
Triglyceride	0.96 (0.71, 1.21)^***^	3.00 (2.39, 3.60)^***^
Glycohemoglobin	1.97 (1.62, 2.33)^***^	5.96 (5.09, 6.82)^***^
Blood urea nitrogen	0.16 (−0.02, 0.33)	0.91 (0.47, 1.35)^***^
Serum creatinine	0.00 (−0.01, 0.02)	0.06 (0.03, 0.10)^***^
Serum uric acid	0.02 (0.02, 0.03)^***^	0.07 (0.06, 0.08)^***^
Total protein (g/L)	−0.04 (−0.12, 0.04)	−0.23 (−0.43, −0.04)^*^

* *P* < 0.05,

** *P* < 0.01,

*** *P* < 0.001.

### Association between urinary nickel and BMI

Table 3 shows the association between urinary nickel and BMI based on multivariate regression analysis. In all three models, no significant associations were found. However, stratified by sex, all three models (model 1: 0.3520, 0.0604–0.6436; model 2: 0.3278, 0.0398–0.6159; model 3: 0.2965, 0.0302–0.5628) revealed a positive association for males, *P* for trend of three models was, respectively, 0.001, 0.003, and 0.010. As a result of secondary stratification based on sex and race, urinary nickel had a positive correlation with BMI in White males (Table 4).

**Table 3:** Association between urinary nickel (ug/L) and body mass index (kg/m^2^).

**Exposure**	**Model 1, β (95% CI)**	**Model 2, β (95% CI)**	**Model 3, β (95% CI)**
urinary nickel (ug/L)	0.1595 (−0.0177, 0.3367)	0.1477 (−0.0283, 0.3237)	0.1243 (−0.0401, 0.2887)
**Stratified by sex**			
Male	0.3520 (0.0604, 0.6436)^*^	0.3278 (0.0398, 0.6159)^*^	0.2965 (0.0302, 0.5628)^*^
**Quintiles of urinary nickel (ug/L)**			
Q1	Reference	Reference	Reference
Q2	−0.0948 (−1.2117, 1.0222)	−0.1758 (−1.2797, 0.9281)	−0.3285 (−1.3483, 0.6913)
Q3	1.3909 (0.2707, 2.5111)^*^	1.2587 (0.1515, 2.3658)^*^	0.8434 (−0.1902, 1.8770)
Q4	1.5478 (0.3398, 2.7558)^*^	1.3839 (0.1870, 2.5808)^*^	1.1568 (0.0419, 2.2717)^*^
*P* for trend	0.001	0.003	0.010
Female	0.0827 (−0.1494, 0.3147)	0.1457 (−0.0304, 0.3217)	0.1032 (−0.0586, 0.2649)
**Quintiles of urinary nickel (ug/L)**			
Q1	Reference	Reference	Reference
Q2	1.2854 (−0.1095, 2.6803)	1.1684 (−0.2166, 2.5534)	0.4477 (−0.7946, 1.6901)
Q3	0.8190 (−0.6159, 2.2540)	0.6397 (−0.7870, 2.0664)	−0.1152 (−1.4114, 1.1809)
Q4	0.7937 (−0.6458, 2.2331)	0.6915 (−0.7433, 2.1263)	−0.4843 (−1.8212, 0.8527)
*P* for trend	0.361	0.452	0.337

Model 1: A covariate adjustment was not made.

Model 2: Adjustments were made for age and race.

Model 3: The variables related to BMI found by univariate analysis in Table 2 were adjusted.

The stratification variable is not taken into account when analyzing subgroups.

* *P* < 0.05.

**Table 4:** Association between urinary nickel (ug/L) and body mass index (kg/m^2^) stratified by sex and race.

**Exposure**	**Model 1, β (95% CI)**	**Model 2, β (95% CI)**	**Model 3, β (95% CI)**
**Male**			
Mexican American	0.1834 (−0.5061, 0.8729)	0.1909 (−0.5014, 0.8831)	0.0914 (−0.5979, 0.7807)
Other Hispanic	−0.9798 (−2.3609, 0.4013)	−0.7445 (−1.9756, 0.4866)	−0.3927 (−1.5063, 0.7209)
Non-Hispanic White	0.6654 (0.1423, 1.1885)^*^	0.5915 (0.0678, 1.1152)^*^	0.5613 (0.0690, 1.0536)^*^
Non-Hispanic Black	0.8468 (0.0592, 1.6344)^*^	0.8986 (0.1172, 1.6800)^*^	0.6527 (−0.0070, 1.3124)
Other race	−0.2210 (−0.6036, 0.1615)	−0.1839 (−0.5650, 0.1972)	−0.1725 (−0.5243, 0.1793)
**Female**			
Mexican American	−0.1073 (−1.0488, 0.8341)	−0.1348 (−1.0795, 0.8099)	−0.2352 (−1.1617, 0.6912)
Other Hispanic	0.8725 (0.0364, 1.7085)^*^	0.8999 (0.0438, 1.7559)^*^	0.8061 (−0.0006, 1.6129)
Non-Hispanic White	0.0135 (−0.4620, 0.4890)	0.0107 (−0.4644, 0.4859)	−0.0921 (−0.5169, 0.3327)
Non-Hispanic Black	0.0424 (−0.2492, 0.3339)	0.0420 (−0.2503, 0.3342)	0.0981 (−0.1701, 0.3663)
Other race	0.0178 (−0.7519, 0.7876)	0.0101 (−0.7613, 0.7815)	−0.2663 (−1.0007, 0.4681)

Model 1: A covariate adjustment was not made.

Model 2: Adjustments were made for age.

Model 3: The variables related to BMI found by univariate analysis in Table 2 were adjusted.

The stratification variable is not taken into account when analyzing subgroups.

* *P* < 0.05.

### Association between urinary nickel and WC

When exploring the association between urinary nickel and WC, we found a positive association in all their models(model 1:0.4894,0.0486–0.9302; model 2:0.4938,0.0679–0.9197; model 3: 0.4110 0.0221–0.7999). However, stratified by sex, the positive association was only found in three male models (model 1: 1.3408, 0.5525–2.1290; model 2: 1.2004, 0.4511–1.9498; model 3:1.1111, 0.4156–1.8066), with a significant *P* for trend of three models (*P* < 0.001, *P* < 0.001, *P* = 0.001) (Table 5). Secondary stratification analysis according to sex and race, urinary nickel has a positive correlation with WC in both White and Black males (Table 6).

**Table 5:** Association between urinary nickel (ug/L) and waist circumference (cm).

**Exposure**	**Model 1, β (95% CI)**	**Model 2, β (95% CI)**	**Model 3, β (95% CI)**
urinary nickel (ug/L)	0.4894 (0.0486, 0.9302)^*^	0.4938 (0.0679, 0.9197)^*^	0.4110 (0.0221, 0.7999)^*^
**Stratified by sex**			
Male	1.3408 (0.5525, 2.1290)^***^	1.2004 (0.4511, 1.9498)^**^	1.1111 (0.4156, 1.8066)^**^
**Quintiles of urinary nickel (ug/L)**			
Q1	reference	reference	reference
Q2	−0.1665 (−3.1852, 2.8523)	−0.0903 (−2.9614, 2.7808)	−0.6539 (−3.3052, 1.9975)
Q3	4.5665 (1.5389, 7.5942)^**^	4.1480 (1.2684, 7.0276)^**^	3.0214 (0.3213, 5.7214)^*^
Q4	5.1487 (1.8838, 8.4135)^**^	4.5536 (1.4405, 7.6667)^**^	3.8424 (0.9421, 6.7428)^**^
P for trend	<0.001	<0.001	0.001
Female	0.1958 (−0.3429, 0.7344)	0.1702 (−0.3608, 0.7012)	0.0899 (−0.3783, 0.5581)
**Quintiles of urinary nickel (ug/L)**			
Q1	Reference	Reference	Reference
Q2	2.9783 (−0.2597, 6.2162)	2.4638 (−0.7253, 5.6529)	1.1570 (−1.6489, 3.9628)
Q3	1.2207 (−2.1101, 4.5516)	1.1005 (−2.1844, 4.3854)	−0.5545 (−3.4614, 2.3524)
Q4	2.0712 (−1.2701, 5.4125)	2.0569 (−1.2468, 5.3606)	−0.2818 (−3.2284, 2.6648)
P for trend	0.368	0.337	0.631

Model 1: A covariate adjustment was not made.

Model 2: Adjustments were made for age, sex, and race.

Model 3: The variables related to WC found by univariate analysis in Table 2 were adjusted.

The stratification variable is not taken into account when analyzing subgroups.

* *P* < 0.05,

** *P* < 0.01,

*** *P* < 0.001.

**Table 6:** Association between urinary nickel (ug/L) and waist circumference (cm) stratified by sex and race.

**Exposure**	**Model 1, β (95% CI)**	**Model 2, β (95% CI)**	**Model 3, β (95% CI)**
**Male**			
Mexican American	0.6573 (−1.0528, 2.3674)	0.7223 (−0.9737, 2.4182)	0.4985 (−1.1143, 2.1112)
Other Hispanic	−2.1927 (−5.9489, 1.5634)	−1.4216 (−4.5441, 1.7009)	−1.0170 (−3.7059, 1.6720)
Non-Hispanic White	2.4342 (0.9895, 3.8789)^**^	1.9892 (0.5901, 3.3883)^**^	1.8745 (0.5521, 3.1969)^**^
Non-Hispanic Black	2.4826 (0.3981, 4.5671)^*^	2.7612 (0.7748, 4.7475)^**^	2.4318 (0.7041, 4.1594)^**^
Other race	−0.2967 (−1.2383, 0.6449)	−0.3579 (−1.3019, 0.5861)	−0.3177 (−1.2222, 0.5868)
**Female**			
Mexican American	-−0.2682 (−2.2340, 1.6976)	−0.4061 (−2.3483, 1.5360)	−0.2736 (−2.1989, 1.6517)
Other Hispanic	1.8316 (−0.0582, 3.7213)	2.1629 (0.2657, 4.0600)^*^	1.7983 (−0.0158, 3.6124)
Non-Hispanic White	−0.1163 (−1.2535, 1.0210)	−0.1326 (−1.2543, 0.9892)	−0.3736 (−1.3291, 0.5819)
Non-Hispanic Black	0.3575 (−0.2903, 1.0052)	0.3620 (−0.2849, 1.0089)	0.4896 (−0.0956, 1.0749)
Other race	−0.2080 (−1.9376, 1.5216)	−0.1975 (−1.9321, 1.5372)	−0.6288 (−2.3407, 1.0830)

Model 1: A covariate adjustment was not made.

Model 2: Adjustments were made for age.

Model 3: The variables related to WC found by univariate analysis in Table 2 were adjusted.

The stratification variable is not taken into account when analyzing subgroups.

* *P* < 0.05,

** *P* < 0.01.

### Association among BMI, WC, and urinary nickel stratified simultaneously by gender and obesity status

As shown in Table 7, when stratified simultaneously according to gender and obesity status, BMI was positively correlated with no obesity in women (0.2161, 0.0325–0.3998), while WC was positively correlated with mixed obesity in Men(1.2598, 0.4131–2.1065).

**Table 7:** Association among body mass index (kg/m^2^), waist circumference (cm), and urinary nickel (ug/L) stratified simultaneously by gender and obesity status.

**Body mass index (kg/m^2^)**	**Male**	**Female**
**Stratified by obsity status**		
No obesity	−0.0107 (−0.2064, 0.1850)	0.2161 (0.0325, 0.3998)^*^
Central obesity	−0.1511 (−0.3176, 0.0154)	0.0204 (−0.1501, 0.1909)
Peripheral obesity	-	-
Mixed obesity	0.2849 (−0.0328, 0.6026)	−0.1046 (−0.3184, 0.1092)
**Waist circumference (cm)**		
**Stratified by obesity status (%)**		
No obesity	−0.0453 (−0.4763, 0.3857)	0.1861 (−0.2251, 0.5973)
Central obesity	0.3738 (−0.1659, 0.9134)	−0.0108 (−0.5076, 0.4861)
Peripheral obesity	-	-
Mixed obesity	1.2598 (0.4131, 2.1065)^**^	−0.0434 (−0.4833, 0.3965)

The variables related to BMI or WC found by univariate analysis in Table 2 were adjusted. The stratification variable is not taken into account when analyzing subgroups.

* *P* < 0.05,

** *P* < 0.01, ^***^P < 0.001.

## Discussion

In this study, urinary nickel was evaluated concerning obesity status in the general population. Our results prove that urinary nickel positively correlates with BMI and WC among adult males but not females. In previous studies, heavy metal pollution is a significant cause of chronic inflammation and oxidative stress, of which nickel occupies a large part (31). Chronic inflammation and oxidative stress can destroy the normal function of cells by interacting. The effects lead to symptoms such as weight gain or loss, decreased libido, physical pain, and emotional disorder, which pose a significant threat to health and lead to chronic inflammatory diseases, including obesity, diabetes, and cancer (32).

Several studies support our findings. Pokorska-Niewiada et al. showed that trace element disturbances, including nickel, can increase body mass index and contribute to endocrine disorders (33). A study from Spain found that the trace element nickel in fat is the highest, highlighting the potential role of nickel in obesity and obesity-related diseases (34). Another study from Turkey directly shows a positive correlation between BMI and nickel (35). The results of Yang et al. proved that men exposed to nickel were more prone to dyslipidemia and BMI ≥ 25 (36). In addition, when Cortés et al. studied the relationship between heavy metal exposure and chronic disease development in Chile, introducing BMI as a variable would confuse the relationship between IL-6 and nickel and increase the impact on individual inflammatory states by 40% at the same time. This study indirectly proves that nickel levels in the urine will affect BMI (37), it indirectly proves that nickel levels in the urine will affect BMI.

When subgroup analyzes were performed, we found that urinary nickel was independently and positively associated with BMI and WC in adult men. Numerous prior research had shown that nickel exposure damages male reproductive organs, which is strongly connected to oxidative stress, DNA damage, and hormonal imbalance (38–40). One study found gender differences in the inflammatory response of mice to the lung after nickel exposure, with the male being more susceptible to acute pneumonia and subchronic lung inflammation than females by a mechanism that induces increased neutrophil by CXCL1 and IL-6/STAT3 signaling pathways and enhanced monocyte infiltration by CXCL1 and CCL2 in male (41). At present, we have not found any other strong evidence for the reason for gender difference related to this study, and we suspect that the reason for the difference may be related to the differences in hormone levels, eating habits, and work stress between men and women. Large-sample prospective studies may be needed to explore this problem.

The precise mechanism of nickel exposure in BMI and WC is still unclear, but we try to clarify it from the following aspects. Firstly, in the hypothalamus, nickel exposure harms neurological function. As a result, hypothalamic neurons degenerate, paraventricular and supraoptic nuclei are reduced, and myeloperoxidase activity, nitric oxide increase, tumor necrosis factor-α and interleukin-1β of factors that promote inflammation ascend, which will affect the endocrine axis and might lead to hormonal imbalances (42, 43); Secondly, there is the possibility that nickel can affect the hypothalamic-pituitary-thyroid axis, causing abnormal thyroid activity (44). Finally, nickel disrupts the function of insulin β cells, resulting in abnormal glucose and lipid metabolism and affecting body weight (45, 46). Nickel exposure has also been linked to diabetes in some studies (20, 47).

Heavy metal contamination is everywhere—vegetables, seafood, meat and poultry, water sources, and household products are all at risk of exceeding heavy metal levels. Long-term nickel exposure causes irreparable harm to human system functioning, yet using nickel-related items in the medical, commercial, and industrial sectors continues to grow fast. The national legislature should reinforce and enhance the pertinent laws and regulations to minimize heavy metal contamination. Our study demonstrated a significant association between nickel exposure and BMI and WC in males, and men with long-term nickel exposure must pay particular attention to this health risk. In addition, the mechanism through which nickel exposure lowers male sperm quality is conclusive, and men with reproductive needs should avoid nickel-related industries. We appeal to the public to reduce exposure to heavy metals, especially nickel.

As a result of the large sample size, valid subgroup analyses were possible. However, some limitations need attention. In terms of screening for overweight and obesity, BMI and WC are highly specific, but they are less sensitive when used to identify adiposity due to their inability to discern fat distribution accurately; higher visceral fat is far more harmful than more fat in areas such as the thighs, and therefore may incorrectly classify a person as unhealthy or at a high-risk category for disease (48). Likewise, a higher BMI may also be induced by increased muscle mass, which may not always indicate obesity (49). Additionally, these two indicators do not account for a multiplicity of characteristics like gender and age. It is well-known that men and women have varying quantities of muscle, which might alter the final indicator findings. Individuals with a high percentage of body fat may create more angiotensin and aldosterone, while muscle does not (50).

## Conclusions

In adult males, both BMI and WC were positively associated with urinary nickel. It is essential for adult men, especially those who are already obese, to reduce their nickel exposure. With the continued growth of nickel applications, nickel-related research will be expanded in the future, and our study may give suggestions for future studies in some specific aspects. Meanwhile, there is a need for further research to understand how urinary nickel might influence BMI and WC.

## Data availability statement

The original contributions presented in the study are publicly available. This data can be found here: www.cdc.gov/nchs/nhanes/.

## Ethics statement

Participation in the study was voluntary, and National Center for Health Statistics Research Ethics Review Board approved the study's conduct. To protect everyone's privacy, NHANES will anonymize collected data before making it public as public data. It is our agreement to follow all guidelines for using NHANES data for research purposes, as well as comply with all applicable standards and laws. The patients/participants provided their written informed consent to participate in this study.

## Author contributions

Conceptualization: H-LL, D-LL, and S-FC. Methodology and Writing—review and editing: G-XW. Software: B-LH. Formal analysis: G-XW and B-LH. Writing—original draft preparation: B-LH. Visualization: J-TL and Z-BF. Supervision: L-YF, H-XZ, H-LL, D-LL, and S-FC. Funding acquisition: S-FC. All authors have read and agreed to the published version of the manuscript.
